# The Genome Sequence of the Eastern Woodchuck (*Marmota monax*) – A Preclinical Animal Model for Chronic Hepatitis B

**DOI:** 10.1534/g3.119.400413

**Published:** 2019-10-23

**Authors:** Tyler S. Alioto, Fernando Cruz, Jèssica Gómez-Garrido, Miriam Triyatni, Marta Gut, Leonor Frias, Anna Esteve-Codina, Stephan Menne, Anna Kiialainen, Nadine Kumpesa, Fabian Birzele, Roland Schmucki, Ivo G. Gut, Olivia Spleiss

**Affiliations:** * CNAG-CRG, Centre for Genomic Regulation (CRG), Barcelona Institute of Science and Technology (BIST), Baldiri i Reixac 4, 08028 Barcelona, Spain,; † Universitat Pompeu Fabra (UPF), Barcelona, Spain,; ‡ Roche Innovation Center Basel, F. Hoffmann-La-Roche Ltd, Basel, Switzerland, and; § Department of Microbiology & Immunology, Georgetown University Medical Center, Washington,

**Keywords:** Eastern Woodchuck, *Marmota monax*, Chronic Hepatitis B, Hepatocellular Carcinoma, Immune Response, Whole Genome Sequencing, Genome Assembly

## Abstract

The Eastern woodchuck (*Marmota monax*) has been extensively used in research of chronic hepatitis B and liver cancer because its infection with the woodchuck hepatitis virus closely resembles a human hepatitis B virus infection. Development of novel immunotherapeutic approaches requires genetic information on immune pathway genes in this animal model. The woodchuck genome was assembled with a combination of high-coverage whole-genome shotgun sequencing of Illumina paired-end, mate-pair libraries and fosmid pool sequencing. The result is a 2.63 Gigabase (Gb) assembly with a contig N50 of 74.5 kilobases (kb), scaffold N50 of 892 kb, and genome completeness of 99.2%. RNA sequencing (RNA-seq) from seven different tissues aided in the annotation of 30,873 protein-coding genes, which in turn encode 41,826 unique protein products. More than 90% of the genes have been functionally annotated, with 82% of them containing open reading frames. This genome sequence and its annotation will enable further research in chronic hepatitis B and hepatocellular carcinoma and contribute to the understanding of immunological responses in the woodchuck.

Approximately 257 million people are chronically infected with hepatitis B virus (HBV) and are at an increased risk of developing cirrhosis and hepatocellular carcinoma (HCC). In 2015 alone, 887,000 people died from HBV-related liver diseases (2017 WHO Global hepatitis report (“WHO | Global hepatitis report, 2017”)).

The majority of individuals with chronic HBV (CHB) currently treated with standard of care (SOC) require long-term or lifelong treatment. SOC such as nucleos(t)ide analogs or interferon effectively suppress viral replication (HBV DNA) but do not eliminate HBV or cure the disease. At present, *functional cure* (defined as sustained, undetectable hepatitis B antigen (HBsAg) and HBV DNA in serum, with or without seroconversion to antibodies against HBsAg) is considered the transformational endpoint for novel HBV therapies (Lok *et al.* 2017). As CHB infection is commonly associated with profound impairment of HBV-specific immune responses, combination of antiviral and immune modulatory therapies will likely be needed to achieve HBV cure. Preclinical testing of new drug candidates in appropriate animal models are necessary for achieving this goal.

Woodchuck hepatitis virus (WHV) is similar to human HBV in its morphology, genome structure, and protein sequence homology. Moreover, WHV infection strongly resembles human HBV infection in terms of pathological and immunological features (Menne and Cote 2007). WHV infection and HBV infection in their respective hosts show age-dependent disease outcomes, including development of HCC. Experimental infection of newborn woodchucks with WHV almost invariably leads to chronic infection (similar to mother-to-child or vertical HBV transmission in humans), whereas animals infected at an older age generally develop acute hepatitis and clinically resolve. Chronic WHV infection in woodchucks usually leads to development of HCC within the first 2-4 years of life.

Thus, the woodchuck represents an attractive animal model to investigate the pathogenesis of HBV infection and virus-induced HCC as well as for testing anti-HBV drug candidates (Tennant *et al.* 2004; Menne and Cote 2007). Indeed, woodchucks have been used extensively over the past three decades in the preclinical evaluation of the efficacy and safety of antiviral compounds for HBV, the majority of which are direct-acting antivirals that target the viral polymerase (Tennant *et al.* 2004; Menne and Cote 2007; Roggendorf *et al.* 2015). New immunotherapies that target immune checkpoints or pathogen recognition receptors to modulate the deficient immune responses in CHB have also been investigated. Preclinical evaluation of agents that activate toll-like receptor 7 (TLR7) or inhibit the programmed cell death protein 1/programmed death-ligand 1 PD/PD-L1 pathway in WHV-infected woodchucks have demonstrated the potential benefits of immunotherapy for HBV cure (Menne *et al.* 2015; Balsitis *et al.* 2018).

However, to fully explore its potential as a preclinical model for testing new HBV drugs, in particular immunotherapy, a better understanding of the woodchuck genome is needed. Recently, cDNAs of the woodchuck immune checkpoint genes T-cell immunoglobulin and mucin-domain containing-3 (Tim-3) and Galectin-9 (LGALS9) have been sequenced and characterized (Liu *et al.* 2017), and the activation of TLRs has been tested (Suslov *et al.* 2018), but knowledge of the genomic sequence of these targets and associated pathways is still lacking.

In this study, we assembled and annotated the genome sequence of the Eastern woodchuck, the results and analysis of which we describe here, including a comparison of relevant gene families to those of humans and mice. This information will serve as the foundation for future research on immune responses and immunomodulation in the woodchuck model.

## Materials and Methods

### Sample Collection

A colony-born WHV-naïve adult female Eastern woodchuck (F6849) was used for genomic DNA isolation from blood and sequencing. Following euthanasia, venous blood was used for the construction of a fosmid library by Lucigen Corp. (Middleton, WI). For the construction of a genomic library, genomic DNA was isolated from blood using the DNeasy Blood and Tissue kit (Qiagen, Valencia, CA) following the manufacturer’s recommendations and eluted and stored in buffer AE of the kit (Table S1).

For transcriptome sequencing, liver, kidney, spleen, lung, and heart from this woodchuck, as well as from other adult WHV-negative woodchucks of both sexes (*i.e.*, F9150, F6852, M4046, M4075, and M4091) were collected (Table S2). Additional thymus and pancreas samples from F9150 were also collected. High molecular weight RNA (> 200bp) was extracted using the RNeasy Mini kit (Qiagen) according to manufacturer’s instructions. Residual genomic DNA was removed using the RNase free DNase set (Qiagen) during the extraction. RNA was quality controlled on Eukaryote total RNA Nano chips (Agilent Technologies). High quality RNA (RIN >7) was obtained for all samples.

### Whole Genome Sequencing

DNA quantity, purity and integrity was verified and aliquots were made for several different library construction protocols. First, three paired-end libraries (471, 589 and 692 bp fragment size) were prepared and sequenced on the Illumina HiSeq2500 platform. The standard Illumina protocol with minor modifications was followed for the creation of short-insert paired-end (PE) libraries (Illumina Inc., Cat. # PE-930-1001). In brief, 2.0 μg of genomic DNA was sheared on a Covaris E220, the fragmented DNA was end-repaired, adenylated and ligated to Illumina-specific PE adaptors. To obtain three PE libraries with approximate fragment sizes of 500 bp, 600 bp and 700 bp, the DNA with adaptor-modified ends was size-selected and purified using the E-gel agarose electrophoresis system (Invitrogen). The PE libraries were run on the HiSeq2500 in 2x150 rapid mode according to standard Illumina operation procedures. A total of 270 Gb of raw sequence (94x coverage) were produced. Primary data analysis was carried out with the standard Illumina pipeline (HCS 2.0.12.0, RTA 1.17.21.3).

Two mate pair (MP) libraries (4 and 7 kb fragment sizes) were constructed according to the Nextera MP preparation protocol, which leaves a linker of known sequence at the junction. Both libraries were sequenced on the HiSeq2000 platform in 2x101 mode, producing 612,944 million pairs (123.8 Gb) of raw sequence for the 4kb library and 518,357 million pairs (104.7 Gb) of raw sequence for the 7kb library.

Additionally, a fosmid library of 155,000 clones was constructed by Lucigen Corp. Ninety-six pools of approximately 1600 clones per pool were made, and the pools were sequenced on the HiSeq2500 in 2x150 rapid mode. Initial estimates indicated an *E. coli* contamination rate of ∼60%, but these reads were removed bioinformatically. In addition, two independent fosmid-end libraries (FE) were constructed by Lucigen and sequenced in two lanes of a HiSeq2000 (2x101), producing 90 Gb of sequence, albeit with a duplicate rate of 88% due to the low complexity of the library. The amount of sequence obtained for each library is summarized in Table 1.

**Table 1:** Output of Sequencing Libraries

library type	library name	insert size	yield mBases	Coverage	avg pct duplicate	avg phix error r1	avg phix error r2
WGS PE	523J_B	471	83996	29.3x	0.33	0.238	0.265
WGS PE	523J_C	589	78061	27.2x	0.27	0.238	0.265
WGS PE	523J_D	692	107291	37.4x	0.26	0.238	0.265
WGS MP	541J-1	3772	123816	43.1x	77.85	0.305	0.308
WGS MP	238L	6782	104709	36.5x	82.21	0.258	0.338
FE	Z047	34543	42194		88.67	0.265	0.280
FE	Z048	34542	47746		87.45	0.265	0.280
FP	pools 1-96*^a^*	333	10608	∼108x	0.86	0.268	0.397

a average values for the 96 fosmid pools.

### RNA Extraction and Sequencing

Sequencing libraries were prepared from either 100 ng or one µg of total RNA using the Illumina TruSeq RNA Sample preparation Kit v2 according to the manufacturer’s instructions. Sequencing libraries were quantified using the Kapa Library Quantification kit (Kapa Biosystems) and quality controlled by capillary electrophoresis on a Bioanalyzer using DNA 1000 chips (Agilent Technologies). Libraries were sequenced on a HiSeq2500 sequencer (Illumina) for 2 × 125 cycles using version 3 cluster generation kits and version 3 sequencing reagents (Illumina). The PhiX control library (Illumina) was spiked into each sample (at 1%) as a sequencing control.

### Genome Assembly

**Figure 1:**
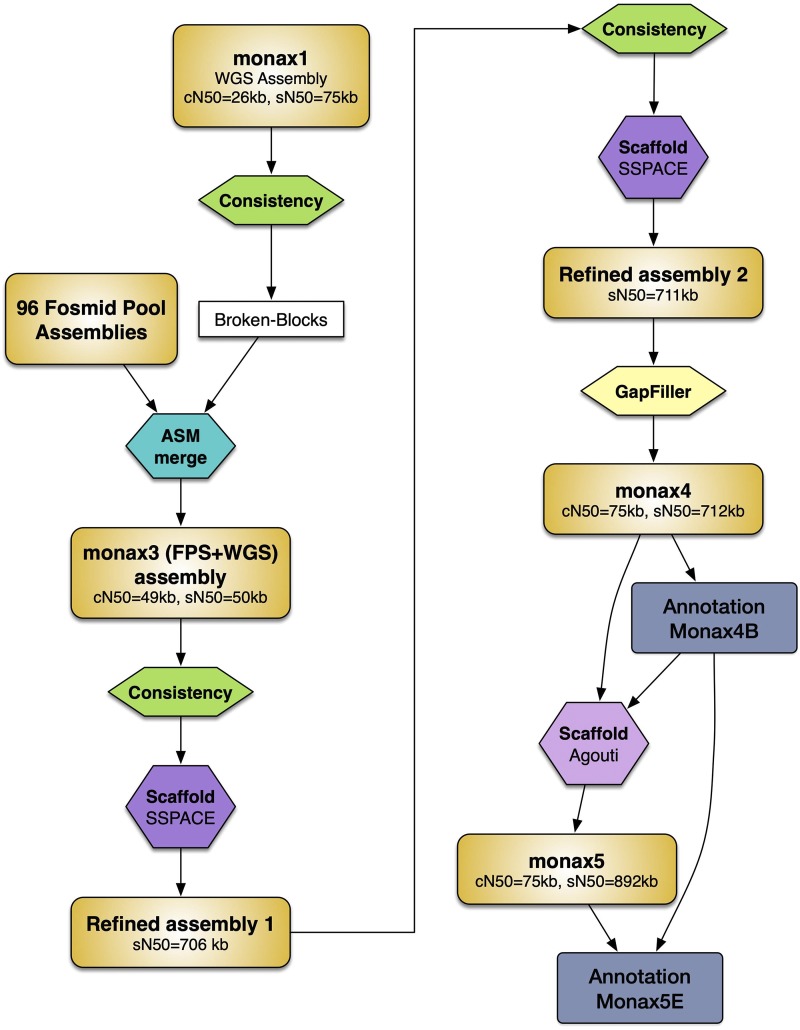
Overview of the assembly workflow. Main assemblies are shown as orange rectangles. Processing steps are shown as colored hexagons. The annotations are represented as blue rectangles.

The assembly steps taken are described here and summarized in Figure 1.

Detection and trimming of Illumina adapter sequences and quality trimming was performed using Trim Galore (https://github.com/FelixKrueger/TrimGalore), which employs the tool *cutadapt* (Martin 2011). The linker sequence present in the MP sequences was also removed with *cutadapt*. Overlapping reads were merged using FLASH (Magoč and Salzberg 2011). Then, all reads were filtered by mapping with gem-mapper (Gemtools v1.6.1: http://gemtools.github.io/) (Marco-Sola *et al.* 2012) with up to 2% mismatches against a contamination database that included phiX, Univec sequences, *E. coli* and the *Marmota himalayana* mitochondrion (NC_018367.1).

To estimate the genome size, an analysis of k-mers present in the sequence reads of all three Illumina PE librarires was carried out using Jellyfish (Marçais and Kingsford 2011) to count k-mers of length 17. A peak k-mer depth was observed at 78-fold k-mer coverage (Fig. S1). A rough estimate of genome size can be made by dividing the total number of counted k-mers (229,897,091,537) by the k-mer coverage (78), which gives 2.95 Gb. Accounting for sequencing error, heterozygosity, and repetitive sequence using the program *gce* (Liu *et al.* 2013), we obtained a more accurate estimate of 2.87 Gb, while GenomeScope v1.0.0 (Vurture *et al.* 2017) estimated a genome size of 2.48 Gb (an underestimate due to filtering of high-copy k-mers) and heterozygosity of 0.24%.

The initial assembly of the three PE libraries using ABySS v1.5.2 (Simpson *et al.* 2009) (with parameters: -s 300 -S 300-5000 -n 8 -N 10 -k 95 -l 50 -aligner map -q 20) had a total length of 2.79 Gb and was characterized by contig and scaffold N50s of 26 kb and 74.5 kb, respectively. Only 0.55% of the assembly corresponded to gaps. This draft assembly (monax1) yielded enough contiguous sequence for estimation of library insert sizes. Mapping of all the sequencing libraries revealed distributions with central peaks close to the expected fragment sizes (Fig. S2).

Subsequently, we assembled each of the 96 fosmid pool (FP) libraries independently. After running some pilot assemblies with ABySS v1.5.7, we determined that the optimal parameters for assembly were k = 85 and l = 75. We used a 12-step pipeline outlined in Fig. S3 and described in (Cruz *et al.* 2016). The resulting fosmid pool assemblies had an average scaffold N50 of 36,920 (s.d. 413), which just exceeds the average insert size determined by mapping the ends back to the assembly (34.5 kb, Fig. S2) itself a lower bound on the true fosmid insert size distribution.

At the same time, we ran a consistency check of the Whole Genome Sequencing (WGS) assembly monax1 using reads from all the whole-genome sequencing libraries (PE, MP and FE). This process introduces breaks at genomic intervals with a negative consistency score (Cruz *et al.* 2016). These consistent WGS contigs were merged with scaffolds from the FP assemblies using the assembly merger ASM (https://github.com/lfrias81/anchor-asm/) with overlap detection edit distance e = 0.02 and mismatch percent m = 0.01. The merged assembly (monax3) was purged of most mis-assemblies by performing another consistency check and re-scaffolded with SSPACE v3.0 (Boetzer *et al.* 2011) and parameters k = 10 and a = 0.6, using the WGS PE, MP and FE libraries and artificially generated MP libraries of fixed insert sizes coming from the FP assemblies. This was followed by another consistency check, re-scaffolding with SSPACE v3.0 and gap-closing with Gapfiller (Boetzer and Pirovano 2012). Contigs smaller than 200 bp were discarded. The resulting assembly (monax4) was substantially more contiguous than the WGS-only assemblies, reaching a scaffold N50 of 712.2 Kb.

### Protein-coding gene annotation

**Figure 2:**
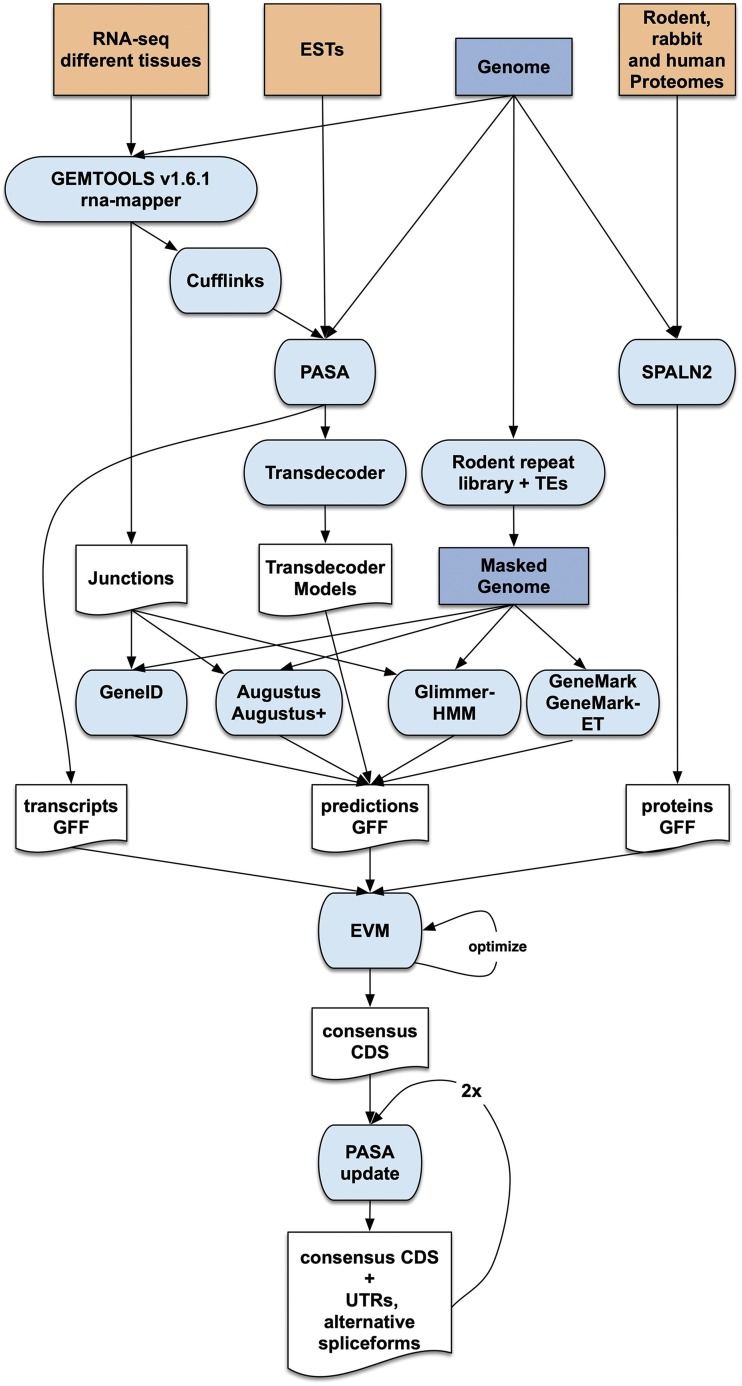
Overview of the protein annotation pipeline. Input data for annotation are shown at the top of the flow chart. Computational steps are shown in light blue and intermediate data are shown in white.

To annotate the woodchuck genome, consensus gene models were obtained by combining transcript alignments, protein alignments and gene predictions with Evidence Modeler (EVM r2012-06-25) (Haas *et al.* 2008). A flowchart outlining these steps is shown in Figure 2.

First, RNA-seq reads corresponding to different tissues and from liver at different time points during chronic viral infection (Fletcher *et al.* 2015) were aligned to the monax4 assembly with GEMtools v1.6.1 rna-mapper (Table 2 and Table S3, respectively) and transcript models were subsequently generated using Cufflinks v2.1.1 (Trapnell *et al.* 2010). These transcripts were then bundled into a non-redundant set by PASA v-2.0.1 (Haas *et al.* 2008). We also ran TransDecoder (Haas *et al.* 2013) to detect coding regions in the transcripts.

**Table 2:** RNA-seq data for tissue samples

Sample	Individual	Tissue	Number of reads	% of reads mapped
**Woodchuck_1**	F6849	Liver	211,888,262	95.113
**Woodchuck_2**	F6849	Kidney	185,982,482	95.506
**Woodchuck_3**	F6849	Spleen	225,337,732	94.303
**Woodchuck_4**	F6849	Lung	206,453,434	91.87
**Woodchuck_5**	F6849	Heart	138,856,972	92.539
**Woodchuck_11**	F6852	Liver	134,795,402	92.029
**Woodchuck_10**	F9150	Thymus	186,450,732	94.791
**Woodchuck_6**	F9150	Liver	144,701,830	94.478
**Woodchuck_7**	F9150	Kidney	179,703,418	93.636
**Woodchuck_8**	F9150	Spleen	142,020,270	89.978
**Woodchuck_9**	F9150	Pancreas	127,666,526	95.619
**Woodchuck_12**	M4046	Liver	202,080,726	94.951
**Woodchuck_13**	M4046	Kidney	127,534,952	93.614
**Woodchuck_14**	M4046	Spleen	125,489,746	94.784
**Woodchuck_15**	M4075	Liver	147,677,126	90.497
**Woodchuck_16**	M4075	Kidney	196,174,530	95.174
**Woodchuck_17**	M4075	Spleen	126,030,468	93.961
**Woodchuck_18**	M4091	Liver	145,226,684	95.157
**Woodchuck_19**	M4091	Kidney	125,790,226	93.04
**Woodchuck_20**	M4091	Spleen	122,753,104	91.042

The complete rodent and rabbit proteomes present in Uniprot (April 8, 2015), as well as the human proteins present in CCDS (April 7, 2015), were aligned to the genome with SPALN v2 (Iwata and Gotoh 2012).

The monax4 assembly was masked for repeats found with RepeatMasker v4-0-5 (http://repeatmasker.org/) using the rodentia library available with the program. Low complexity repeats were left unmasked for this purpose. In addition, we were able to mask additional transposable elements (TEs) in the genome via a separate Basic Local Alignment Search Tool (BLAST) (Altschul *et al.* 1990) search of proteins encoded by TEs (downloaded from RepBase).

Then, *ab initio* gene predictions were performed on the masked assembly. Four different gene prediction programs were used: GeneID (Parra *et al.* 2000; Alioto *et al.* 2018), Augustus (Stanke and Waack 2003), GeneMark-ET (Lomsadze *et al.* 2005) and Glimmer (Majoros *et al.* 2004). GeneID *ab initio* gene predictions were obtained by running GeneID v1.4 with the pre-existing parameter file specific for *Homo sapiens* that has been previously used to accurately generate gene predictions in several different mammalian genomes (Venter *et al.* 2001; Mouse Genome Sequencing Consortium *et al.* 2002; Gibbs *et al.* 2004; Bovine Genome Sequencing and Analysis Consortium *et al.* 2009; Abascal *et al.* 2016; Hernandez *et al.* 2019). Augustus v3.0.2 and Glimmer Hidden Markov Model (HMM) v3.0.1 were also run with the program’s pre-existing human parameter file while GeneMark-ES v2.3e gene predictions were obtained using its self-training mode. The number of predicted gene models ranged from 33,959 with GlimmerHMM to 73,562 with GeneID.

GeneID, Augustus and GeneMark-ET were also used to generate predictions incorporating intron evidence, which was extracted from the RNA-seq mappings. Those junctions overlapping *ab initio* GeneID predictions, Augustus predictions or protein mappings were taken as intron evidence.

The assembled transcripts, the protein alignments and the models produced by Glimmer, GeneID, Augustus and GeneMark-ES were combined into consensus CDS models using EvidenceModeler (EVM). Different weights were given to each type of evidence when running EVM and the resulting consensus models with the best specificity and sensitivity as determined by intersection (BEDTools v 2.22.1 (Quinlan and Hall 2010)) with the transcript mappings were chosen for the final annotation (final weights given in Table S4).

The consensus CDS models were then updated with untranslated regions (UTRs) and alternative exons through two rounds of PASA annotation updates. A final round of quality control was performed, fixing reading frames, intron phases and removing transcripts that would be subject to nonsense-mediated decay (NMD). The resulting transcripts were clustered into genes using shared splice sites or significant sequence overlap as criteria for designation as the same gene. Systematic identifiers with the prefix “MONAX4B” were assigned to the genes, transcripts and protein products derived from them. Support by source of evidence at the gene and exon level was determined *a posteriori* using BEDTools *intersect* and *multiinter* programs.

Finally, this resulting annotation (Monax4B), along with RNA-seq mappings produced by STAR v2.5.0b (Dobin *et al.* 2013) with the option “–chimSegmentMin 40” to allow chimeric mappings between different scaffolds, was used to scaffold the monax4 assembly with Agouti to produce the final monax5 assembly. Some partial genes previously located in separate scaffolds were also able to be joined. After obtaining this new version of the assembly, the coordinates of the annotation were transferred to monax5 scaffolds and the genes were given “MONAX5” prefixes.

### Functional annotation

To assign a functional description to the structurally annotated genes, we ran the software Trinotate (Bryant *et al.* 2017) (r20140708), which performs BLAST (Altschul *et al.* 1990) searches against the Swissprot and Uniprot databases and detects Pfam (Punta *et al.* 2012) domains in the annotated proteins using HMMER (Finn *et al.* 2011). Finally, the outputs of all the previous steps were combined into the Trinotate sqlite database to generate the functional descriptions, functional domains and gene ontologies (Ashburner *et al.* 2000) for each gene. Of the 44,630 transcripts, 40,036 are supported by protein alignments, 34,601 by PASA assemblies, and 42,476 (95%) by either protein or PASA transcript assemblies. The remaining transcripts are supported only by *ab initio* gene predictions. Also, 27,831 out of the 30,873 (90%) protein coding genes were functionally annotated, 25,192 had at least one associated GO term, and 13,186 were assigned to an eggnog orthologous category.

### Non-coding RNA annotation

We annotated non-coding RNAs (ncRNAs) by running the following steps. First, the program *cmsearch* that comes with Infernal v1.1 (Nawrocki and Eddy 2013) was run with the Rfam database of RNA families (v12.0) (Nawrocki *et al.* 2015). Also, tRNAscan-SE v1.23 (Lowe and Eddy 1997) was run in order to detect the transfer RNA genes present in the genome assembly. To detect long non-coding RNAs (lncRNAs) we first selected PASA-assemblies that had not been included in the annotation of protein-coding genes, *i.e.*, expressed genes that were not translated to protein. Those that were longer than 200bp and whose length was not covered at least 80% by a small ncRNA were incorporated into the ncRNA annotation as lncRNAs. The resulting transcripts were clustered into genes using shared splice sites or significant sequence overlap as criteria for designation as the same gene.

### Gene Expression

The RNA samples of thymus, heart, pancreas, kidney, spleen and liver used for annotation were also quantified. RNA-seq reads for each sample/replicate were mapped against the woodchuck genome assembly using the gem-tools rna-mapper v1.6.1 with default options and transcripts were quantified with gemtools version 1.7.1 using the Monax4B annotation. Normalization of gene expression was done with the TMM method (Robinson and Oshlack 2010) of edgeR software. Differential expression was determined using the edgeR robust approach (Zhou *et al.* 2014).

### Mitochondrial Sequence Analysis

We assembled the complete circular mitochondrial chromosome of the woodchuck as follows. Read pairs from the 700bp-insert 523J_D paired-end library mapping to the Himalayan marmot mitochondrial sequence (read filtering step above) were downsampled (10,000 reads) and assembled with SPAdes v3.13.0 (Bankevich *et al.* 2012), which resulted in a scaffold of length 15.6 kb containing four gaps. Novoplasty v2.5.7 (Dierckxsens *et al.* 2017) was used to complete the assembly using all reads of the 523J_D library with the SPAdes scaffold as the seed. This resulted in a single contig with a length of 16440bp. It is 92.78% and 92.75% identical to the himalayan (NC_018367.1) and European (NC_027278.1) alpine marmot mitochondria, respectively, while the European and Himalayan marmot sequences are slightly more similar to each other (92.96% identical).

### Orthology determination

To determine the orthology/paralogy relationships between all woodchuck proteins and those of human and mouse we ran Orthoinspector v2.14, which took as input the results of an all-*vs.*-all BLAST search of all the proteins in the three genomes against themselves. For a more limited set of genes, we obtained the phylogenetic trees and corresponding multiple sequence alignments (MSAs) for each of them from PhylomeDB (http://phylomedb.org/). We then extracted their candidate orthologs in the woodchuck genome from the Orthoinspector output. As the *Marmota marmota marmota* genome was also available, we also included it in the trees: we downloaded the proteins from NCBI (PRJEB8272) and performed a BLAST search of each human protein sequence of interest against the *Marmota marmota marmota* proteins and selected those hits of e-value less than 10^−9^. Next, we aligned the woodchuck and *Marmota marmota* sequences to the existing multiple sequence alignment. The alignment was performed with MUSCLE (Edgar 2004) and trimmed with TrimAl (Capella-Gutiérrez *et al.* 2009). Next, phylogenetic trees for each alignment were constructed with PhyML v3 (Criscuolo 2011), testing four different models (WAG, JTT, Blosum62 and VT) and selecting the one with the maximum likelihood. Finally, we determined the paralogous and orthologous relationships based on the tree topologies. Gene family trees were constructed and visualized with the ETE 3 toolkit using the “full_fast_modeltest_bootstrap” workflow.

### Data availability

The DNA sequence reads, the genome assembly and the annotation have been deposited in the European Nucleotide Archive (ENA) with the project accession (PRJEB19462). The RNA-seq data from this publication have been submitted to NCBI’s GEO database (http://www.ncbi.nlm.nih.gov/geo) and assigned the identifier GSE137911. Also, in order to facilitate access to the genome as a resource, we have made available a genome browser and a BLAST server at http://denovo.cnag.cat/woodchuck. Supplemental material available at figshare: https://doi.org/10.25387/g3.10013024.

## Results

### Genome Assembly

The final 2.62 Gbp assembly (monax5) is made up of 48,534 scaffolds with a scaffold N50 of 892kb. Genome completeness was determined using both CEGMA v2.5 (Parra *et al.* 2007), which found 96.4% complete and 2.8% partial genes out of 248 core eukaryotic genes, and Benchmarking Universal Single-Copy Orthologs (BUSCO) v3.0.2 (Simão *et al.* 2015), which found 93% complete and 3.7% partial genes using the mammalian odb9 library (4104 genes). Statistics for all major stages of the assembly process are given in Table 3.

**Table 3:** Summary statistics of major assembly steps

	Contiguity						Gene Completeness	
	Contigs			Scaffolds			CEGMA	
	N50 (kb)	N90 (kb)	Length (Gb)	N50 (kb)	N90 (kb)	Length (Gb)	Complete (%)	Partial (%)
*monax*1	26.1	3.4	2.79	74.6	8.7	2.79	88.71	98.39
*monax*3	48.8	9.1	2.54	49.5	9.2	2.55	*^a^*	*^a^*
*monax*4	74.5	15.6	2.55	712.2	112.8	2.62	96.37	99.19
*monax5*	74.5	15.6	2.55	892.2	124.4	2.63	96.37	99.19

a gene completeness not determined for assembly monax3.

### Protein-coding gene annotation and expression

The protein-coding gene annotation (Monax5E) led to a set of 30,873 protein-coding genes whose 44,630 transcripts encode 41,826 unique protein products (∼1.45 transcripts per gene) (Table 4).

**Table 4:** Protein-coding annotation statistics

	Monax5E
**Number of genes**	30873
**Median Gene length (bp)**	6123
**Number of transcripts**	44630
**Number of proteins**	41826
**Number of partial CDS**	6232
**Number of exons**	217018
**Number of coding exons**	204230
**Transcripts per gene**	1.45
**Exons per transcript**	6.1
**Multi-exonic transcripts**	0.73
**Coding GC content**	52.81%

**Figure 3:**
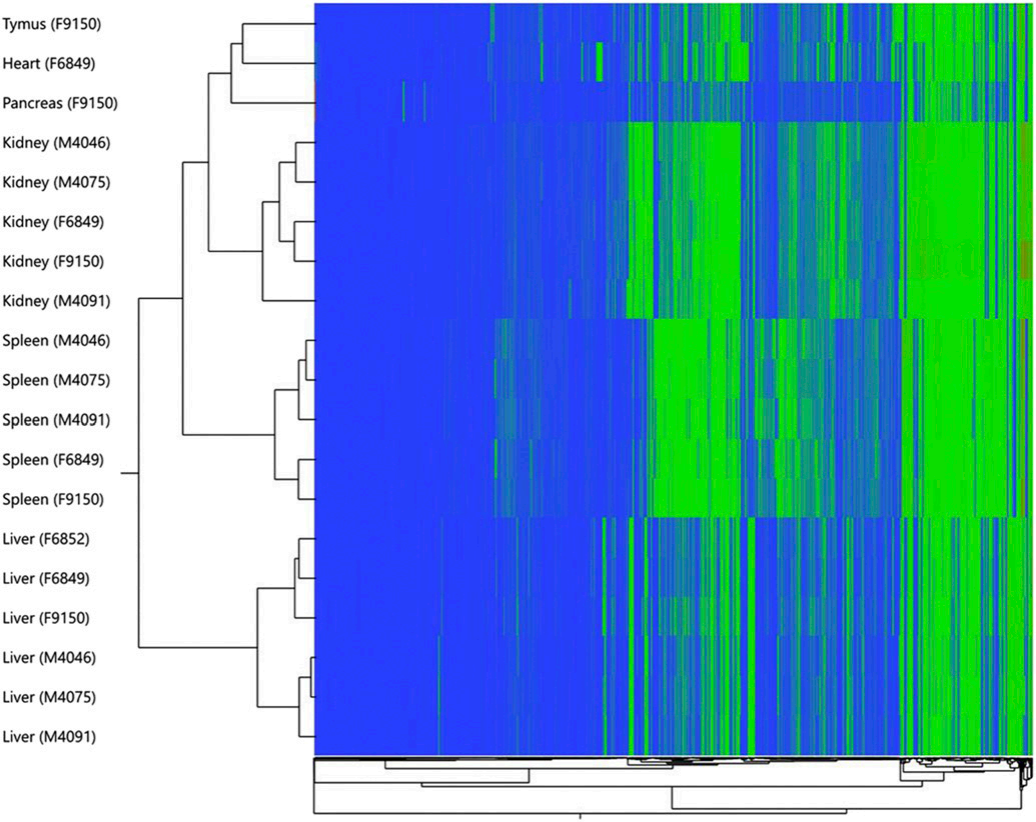
Hierarchical clustering of RNA-seq gene expression levels in six tissues.

While RNA-seq scaffolding with Agouti was able to join some genes that were originally located in separate scaffolds, about 6000 genes remain without complete open reading frames, suggesting that a number of genes are only partial and that the true number of genes in the genome is likely lower. Hierarchical clustering of the RNA-seq samples (Figure 3) shows high similarity of expression obtained for each tissue across individuals compared to cross-tissue similarity in single individuals, as would be expected.

### Phylogenetic Analysis of Immune Genes

We identified human-mouse-woodchuck orthologs and in-paralogs using Orthoinspecter, a best reciprocal BLAST-based ortholgoy assignment method. We obtained a slightly higher number of one-to-one orthologs with woodchuck-to-human compared to mouse-to-human, with a corresponding decrease in one-to-many in-paralog relationships, as shown in Table 5.

**Table 5:** Orthoinspector results. A pairwise comparison of the number of one-to-one orthologs or in-paralogs detected by Orthoinspector among human, mouse and woodchuck protein products. Out-paralogs are not shown

	*One-to-one*	*One-to-many*	*Many-to-one*	*Many-to-many*
***Human-Woodchuck***	11,481	1,788	2,054	485
***Mouse-Woodchuck***	12,291	1,018	3,102	892
***Human-Mouse***	10,535	3,444	1,540	749

The highest number of one-to-one orthologs is found between mouse and woodchuck, which is not surprising, but the number of many-to-one and one-to-many in-paralog relationships is not balanced, with mouse exhibiting more gene family expansions. The same can be observed for mouse *vs.* human, whereas gene family expansions appear more balanced between woochuck and human.

However, programs based on BLAST searches, such as Orthoinspector, have limitations and do not always correctly determine orthologs and paralogs. Phylogenetic methods are more robust, but they are also more computationally intensive (Huerta-Cepas *et al.* 2007). With this in mind, we compiled a set of 40 human genes (see Table S5**)** involved in the immunological response to HBV or liver cancer and constructed phylogenetic trees for this more limited set to confirm their orthology to predicted woodchuck gene products. The complete list of woodchuck genes and their orthologs in mouse and human is given in Table S5.

As many members of the Interferon (IFN) and Toll-like receptor families were present in our list of genes of interest, we also constructed global gene family trees (Fig. S4 and Fig. S5). Comparison of our IFN gene tree topology to the one in PhylomeDB (http://phylomedb.org/?q=search_tree&seqid=IFNA) suggests that the duplications of the IFNA (15 genes) and IFNL (4 genes) genes, although being present in human, mouse and woodchuck, took place independently in each branch. However, our data suggests that the main expansion in marmots took place before the speciation event that separated woodchuck and the alpine marmot, although it probably continued in the woodchuck lineage (as we only found 2 INFL and 10 IFNA genes in M. m*armota marmota*). This observation is concordant with the slow rate of evolution observed in the alpine marmot nuclear genome (Gossmann *et al.* 2019). Moreover, we detected a duplication of the IFNB gene in the marmot lineage that is not present in human or mouse. However, we failed to identify an ortholog of the IFNK gene in our annotation, although we did find one in the alpine marmot. Further research is needed to determine whether this gene has really been lost in the woodchuck or is just missing in our assembly.

In the TLR family tree, we were able to assign one-to-one relationships between all the genes in human, woodchuck and alpine marmot, except for TLR2, for which we have found a partial duplication in the *M. monax* genome.

## Conclusion

The woodchuck genome assembly will be a valuable resource for further investigation of viral hepatitis and HCC in the woodchuck model. The availability of new gene expression data from several tissues together with the genome sequence further increases the value of the woodchuck model for human drug development. Specifically, it will generate more insight into immune response pathways and aid the characterization of important genes in the woodchuck immune system.
